# A global study into Indian women’s experiences of domestic violence and control: the role of patriarchal beliefs

**DOI:** 10.3389/fpsyg.2024.1273401

**Published:** 2024-03-01

**Authors:** Lata Satyen, Madeleine Bourke-Ibbs, Bosco Rowland

**Affiliations:** School of Psychology, Deakin University, Melbourne, VIC, Australia

**Keywords:** domestic violence, patriarchal beliefs, control, feminist framework, Indian communities

## Abstract

Domestic violence (DV) is a serious and preventable human rights issue that disproportionately affects certain groups of people, including Indian women. Feminist theory suggests that patriarchal ideologies produce an entitlement in male perpetrators of DV; however, this has not been examined in the context of women from the Indian subcontinent. This study examined Indian women’s experiences of abuse (physical, sexual, and psychological) and controlling behavior across 31 countries by examining the relationship between the patriarchal beliefs held by the women’s partners and the women’s experience of DV. This study uses an intersectional feminist framework to examine the variables. Data from an online questionnaire was collected from 825 Indian women aged between 18 and 77 years (*M* = 35.64, *SD* = 8.71) living in 31 countries across Asia (37.1%), Europe (18.3%), Oceania (23.8%), the Americas (16.1%) and Africa (3.2%) and analyzed using a hierarchical linear regression. A majority of participants (72.5%) had experienced at least one form of abuse during their relationship, and over a third (35.1%) had experienced controlling behavior. In support of the central hypotheses, after controlling for potential confounders, women whose partners showed greater endorsement of patriarchal beliefs were less likely to have access to freedom during their relationship (*ß* = −0.38, *p* < 0.001) and were more likely to have been abused by their partner or a member of his family (*ß* = 0.34, *p* < 0.001). The findings of this study highlight the need to engage with men in Indian communities through culturally-tailored intervention strategies designed to challenge the patriarchal ideologies that propagate, justify, and excuse DV.

## Introduction

Domestic violence (DV) is the most common form of violence against women, and occurs in every country around the world, transgressing social, economic, religious, and cultural divides (García-Moreno et al., 2005; Violence Against Women Prevalence Estimates, 2018). Although men can be abused by female partners and violence also occurs in non-heterosexual relationships, the vast majority of DV victims are women, and their perpetrators are a current or former male partner (World Health Organization, 2019). In the context of this study, DV includes physical, sexual abuse, or emotional abuse and controlling behaviors such as enforced isolation, excessive jealousy, and limiting access to economic resources or support (Our Watch, 2015; World Health Organization, 2019). In research, the terms, domestic violence, intimate partner violence, family violence, sexual violence and spousal abuse are used interchangeably. For the purposes of the present study, ‘domestic violence’ is used to refer to the violence women experience from their current or former intimate partner.

In addition to representing the leading cause of death for women around the world, with more than 50,000 women being killed by a partner or family member each year (UNODC, 2018), the physical, psychological, and social effects of DV are profound and enduring. Along with physical injuries, women who have been subjected to DV report higher rates of depression, anxiety disorders, post-traumatic stress disorder, cognitive impairment, substance abuse, and are more likely to have thought about or attempted suicide (Ellsberg et al., 2008; Chandra et al., 2009). They are also at a heightened risk of experiencing sexually-transmitted infections, gynecological problems, unwanted pregnancies, and miscarriages (Ellsberg et al., 2008; Stephenson et al., 2008; Dalal and Lindqvist, 2010). Moreover, violence in the home places women at significant risk of homelessness, unemployment, and poverty (Specialist homelessness services annual report, Summary, 2021). Although some men also experience violence from their female partners, prevalence rates from across the world show that women experience violence at three times a greater rate than men; the risk factors for men and women could also vary and therefore, these need to be clearly delineated for each group. Given the deleterious outcomes associated with DV, understanding the factors that drive it is vital in research, policy, as well as in clinical practice (Ellsberg et al., 2008).

A landmark study by the WHO which collected data from over 24,000 women in 10 countries about the extent of domestic violence they experienced found that depending on country and context (e.g., rural versus urban locations), between 15 and 71% of women had been physically or sexually assaulted by an intimate partner during their lifetime (García-Moreno et al., 2005). These findings raise three pertinent points: first, that the apparent universality of DV confirms that its occurrence is not a random aberration, but instead a reflection of gender inequalities that are deeply entrenched and systemically enacted in many cultures and societies around the world. Second, that in addition to gender, factors such as socioeconomic status, ethnicity, and immigration status intersect with gender to shape women’s experiences of abuse. Third, that high rates of violence against women are not inevitable, nor intractable, and therefore should be the aim of global prevention efforts. In sum, it is clear that the harmful effects of DV are universal, but not experienced by all women equally. As such, identifying how diverse groups of women experience DV in their particular cultural context is essential for designing culturally relevant interventions for both victims and perpetrators (Bhuyan and Senturia, 2005). Studies have shown that the experiences of migrant and refugee women can vary significantly to their non-migrant counterparts, therefore, we need a clearer understanding of the nuances of these differences and the impacts of their experiences.

Indian women are one group of women that remain at high risk of DV with or without migration from India (Natarajan, 2002; Ahmed-Ghosh, 2004; Bhuyan and Senturia, 2005) compared to women from Europe, the Western Pacific or North America (Violence Against Women Prevalence Estimates, 2018). However, the largely Western-centric feminist discourse surrounding DV means there is a dearth of Indian-specific research. In addition, common methodological limitations such as the lack of psychometrically-validated, culturally-appropriate DV measurement tools, small and single-location sample sizes, and a failure to recognize forms of abuse other than physical abuse means that the voices of Indian women remain both under-and mis-represented in the extant literature (Yoshihama, 2001; Kalokhe et al., 2016).

While much progress has been made toward gender equality in India (Bhatia, 2012), the prevalence of DV is high. Data from the 2015–2016 Indian National Family Health Survey indicated that 33% of the 67,000 women surveyed in India had experienced DV during their marriage, with the most common type being physical violence (30%), followed by emotional (14%) and sexual violence (7%) (National Family Health Survey, 2017). A recent systematic review of 137 quantitative studies examining DV in India by Kalokhe and colleagues (Kalokhe et al., 2016) also found high rates of these types of violence along with a 41% prevalence of multiple types of abuse. The impact of physical, sexual, and psychological abuse on women’s mental, physical, sexual, and reproductive health is severe and leads to greater levels of depression, suicide attempts, post-traumatic stress disorder, and somatic symptoms and a decreased quality of life (Kalokhe et al., 2016). Research also shows that Indian women who have migrated from India to the United Kingdom, the United States, and Canada experience higher rates of DV than the general population (Raj and Silverman, 2002; Ahmad et al., 2004; Mahapatra, 2012). Little is known about the DV rates among Indian women who migrate to other countries. Taken together, these findings suggest that Indian women across the globe experience high rates of DV. As such, it is important to understand the sociocultural factors that contribute to its occurrence.

While there is no single cause of DV, feminist theories emphasize how the circulation and espousal of patriarchal ideologies in society contribute to, create, and maintain DV (Pagelow, 1981; Smith, 1990). Although variously defined, patriarchy refers to the hierarchical system of social power arrangements that affords men more power and privilege than women, both structurally and ideologically (Smith, 1990; Hunnicutt, 2009) with the origins of the word ‘patriarchy’ coming from the Greek word *Πατριάρχης* (*patriakh͞es*), meaning male chief or head of a family.

According to an ecological framework (Heise, 1998), patriarchal control, exploitation and oppression of women occurs within all levels of social ecology, including the macrosystem (e.g., government, laws, culture), mesosystem (e.g., the media, workplaces), microsystem (e.g., families and relationships), and at the level of the individual. Through social learning, patriarchal structures are internalized as patriarchal ideologies, which are a set of beliefs that legitimize and justify the expression of male power and authority over women, including DV (Smith, 1990; Yoon et al., 2015). More specifically, patriarchal beliefs include notions about the inherent inferiority of women and girls, men’s right to control decision-making in both public and private spheres, traditional and proscriptive gender roles, and the condoning of violence against women (Our Watch, 2015; Yoon et al., 2015). Such ideologies preserve and strengthen the structural gender inequalities that set the necessary social context for DV to occur, by giving men the cultural, legal, and social mandate to use varying degrees of violence and control against women (Our Watch, 2015; Yoon et al., 2015; World Health Organization, 2019).

Research from the United States indicates that positive attitudes toward violence against women and beliefs in traditional gender roles is associated with perpetration of DV (Sugarman and Frankel, 1996; Stith et al., 2004). Similarly, Hah-Yahia (Haj-Yahia, 2005) found that Jordanian men who subscribed to patriarchal ideologies were more likely to justify DV, blame women for violence against them, believe that women benefit from beating, and believe that men should not be punished for hurting their wives. Furthermore, a study of South Asian women living in the United States found that women who endorsed patriarchal beliefs were more likely to have experienced DV (Adam and Schewe, 2007), and men in Pakistan who adhered to patriarchal ideology were more likely to use physical violence against their partners (Adam and Schewe, 2007).

Despite its clear theoretical underpinnings, the relationship between patriarchal beliefs as a single construct and DV perpetration in Indian communities has, to the best of the authors’ knowledge, not been quantitatively examined. This is important, as although patriarchy is omnipresent in all societies on earth, culture shapes its manifestation through values, norms, beliefs, traditions, and familial roles that perpetuate patriarchal structures and ideologies (Duncan, 2002).

In Indian families, power and authority is transmitted from father to the eldest son, meaning that females are expected to be subservient to males throughout their lifetimes; in childhood, to their fathers; upon marriage, to their husbands; and in old age (on occasion of the death of their husband), to their sons (Bhuyan and Senturia, 2005). The impact of a father’s violence on children’s development can last a long time. Research suggests that the effects of this violence against girls in childhood are much more serious and deleterious than the effects of violence used by other men, or even a mother, against women such that women who suffer violence by their father have low levels of resilience in adulthood – even though they might report other perpetrators (such as the husband) as committing greater violence (Tsirigotis and Łuczak, 2018). Therefore, women, as adults, can continue to be affected by patriarchal behaviors of men. In the Indian context, historically too, the hierarchy between men and women prevailed. For example, in ancient India, Smriti, Kautilya, and Manu philosophers demanded total subservience of women to their husbands (Kumar, 2017). In spite of advances in society about gender equality and gender roles, such attitudes still exist in India. For instance, the Indian National Family Health Survey found that less than two-thirds, that is, 63% of married women participated in decision-making about major household matters, and less than 41% were allowed to go to places such as the market, a health facility, or visit relatives alone (National Family Health Survey, 2017).

Prescriptive gender roles contribute to the incidence of domestic violence by positioning women as subordinate, with men therefore tasked with ‘protecting’ women and ensuring they uphold the gendered expectations and moral standards imposed on them (Haj-Yahia, 2005; Satyen, 2021). Indeed, physical violence is viewed as a common and acceptable response to women’s “disobedience,” or failure to meet her husband’s expectations (Jejeebhoy and Cook, 1997). For example, 42% of men and 52% of women believed that a husband is justified in beating his wife if she goes outside without telling him, neglects the house, argues with him, refuses to have sex, does not cook properly, is suspected of being unfaithful, or is disrespectful. This demonstrates that women have possibly internalized their “inferior” status in society and are more accepting of the inequality they face in the household. Honor killings, where women are killed by male family members for bringing shame to their families, still occurs in India and may represent the most extreme example of such attitudes (Kumar and Gupta, 2022).

Taken together, the aforementioned findings clearly outline the broad links between DV and elements of patriarchal ideology including ideas about the inherent inferiority of women, men’s right to control decision-making, traditional gender roles, and condoning of violence against women (Our Watch, 2015; Yoon et al., 2015). However, lacking from this literature is a culturally-specific, comprehensive assessment of the role of individual-level patriarchal beliefs in influencing Indian women’s experiences of DV. Understanding this relationship is vital in order to develop culturally tailored DV interventions and policies.

While cultural expressions of patriarchy provide the necessary context for DV to occur, according to intersectionality theory (Kumar and Gupta, 2022), gender oppression intersects with other forms of inequality, such as poverty, racism, and migration status to increase the risk of DV for certain groups of Indian women (Sokoloff and Dupont, 2005). For example, those who are younger, have more children, live in rural locations, have fewer years of schooling, or who are unemployed are more likely to experience DV during their lifetime (Sokoloff and Dupont, 2005), and may be less likely to seek help for DV (Leonardsson and San, 2017). Furthermore, migration has been identified as a key risk factor for DV (Satyen et al., 2018; UNODC, 2018; Satyen, 2021), through practical and cultural barriers to accessing help and support (Raj and Silverman, 2002; Colucci et al., 2013), as well as so-called ‘backlash’ factors, whereby men increase their use of violence and control following migration to more egalitarian locations, in response to the threatened loss of status and authority (Dasgupta and Warrier, 1996; Zavala and Spohn, 2010). In examining DV, it is therefore important to acknowledge the compounding effects of such factors, while underscoring the central role of patriarchy (Gundappa and Rathod, 2012).

The objective of this study was to examine Indian women’s experiences of abuse (physical, sexual, and psychological) and controlling behavior across 31 countries by examining the relationship between the patriarchal beliefs held by the women’s partners and the women’s experience of DV. Given our understanding of how patriarchal beliefs relate to DV, it was hypothesized that a greater endorsement of patriarchal beliefs by a woman’s partner would predict greater occurrence of abuse and controlling behavior during their relationship.

## Method

### Research design

We examined the relationship between women’s partners’ patriarchal beliefs (as reported by the women) and the women’s experiences of DV using an intersectional feminist lens. This study used a quantitative, cross-sectional design using an online survey, which explored the impact of partners’ patriarchal beliefs on Indian women’s experiences of DV. The inclusion criteria for partaking in the study included: women who identified culturally as belonging to or having origins in the Indian sub-continent. They needed to have been in the past or currently be in an intimate partner relationship. They could be living in the Indian sub-continent or have migrated elsewhere in the world. They needed to also be 18 years and over to take part in the study and have minimal English language skills to comprehend the questionnaires.

### Participants

Participants for this study were recruited from across the world via social media and culturally relevant organizations. Through targeted recruitment, Indian women 18 years or over who were currently in or had previously been in an intimate relationship with a male were asked to participate in the study. In addition to recruiting from India and Australia, data from the Government of India’s Ministry of External Affairs (Population of Overseas Indians, 2018) was used to identify the 15 countries with the highest population of people of Indian origin and these were targeted for recruitment in addition to promoting the study across other countries. Target countries included: the United States, United Arab Emirates, Malaysia, Saudi Arabia, Myanmar, the United Kingdom, Sri Lanka, South Africa, Pakistan, Canada, Kuwait, Mauritius, Qatar, Oman and Singapore. In total, 349 organizations and community groups were contacted by email and provided details of the study. Further, A Facebook page was set up for the project, and a recruitment advertisement was posted to 1,167 public groups relating to Indian women’s interests. In all, 825 participants aged between 18 and 77 years (*M* = 35.64, *SD* = 8.71) from 31 countries across Asia (37.1%), Europe (18.3%), Oceania (23.8%), the Americas (16.1%) and Africa (3.2%) took part. The majority of them were born in India (*n* = 720, 87.3%), but 59.3% had migrated from their country (India or other) of birth. See Table 1 for a detailed summary of their demographic characteristics.

**Table 1:** Demographic characteristics of the sample (*N* = 825).

Variable	Frequency (*n*)	Percentage (*%*)
Age (*M* = 35.65, *SD* = 8.71) 18–30 31–40 41–50 51–60 > 60	237 405 132 38 13	28.7 49.1 16.0 4.6 1.6
Continent of origin Oceania Asia Europe Americas Africa	11 747 20 10 37	1.3 90.5 2.4 1.2 4.5
Continent of residence Oceania Asia Europe Americas Africa	196 306 151 133 26	23.8 37.1 18.3 16.1 3.2
Migration status Migrant Non-migrant	489 334	59.5 40.5
Religion Buddhist Catholic Other Christian Hindu Sikh Muslim Other Atheism	5 57 35 546 63 31 21 64	0.6 6.9 4.2 66.2 7.6 3.8 2.5 7.8
Marital status Married Divorced or separated *De facto* Widowed	649 124 41 9	78.7 15.0 5.0 1.1
Living arrangements Alone With partner With children With natal family With partner’s family Non-family members	43 480 485 80 104 40	5.2 58.2 58.8 9.7 12.6 4.8
No. of children (*M* = 1.06, *SD* = 0.89) Zero One Two Three Four or more	232 289 216 21 6	28.1 35.0 26.2 2.6 0.7
Highest level of education Primary Secondary Vocational Undergraduate Postgraduate Other	6 21 10 239 495 51	0.7 2.5 1.2 29.0 60.0 6.2
Employment status Unemployed Part-time Full-time Casual Volunteer	246 113 397 35 33	29.8 13.7 48.1 4.2 4.0
Partner’s employment status Unemployed Part-time Full-time Casual	53 33 688 22	6.4 4.0 83.4 2.7
Compared to partner, earns More About the same Less	140 128 516	17.0 15.5 62.5

### Measures

Participants completed an online questionnaire that assessed demographic information, their experiences of domestic violence, and their partners’ patriarchal beliefs.

#### Demographic information

Participants’ age, country of birth, country of residence, migration status, religion, marital status, and educational attainment were collected.

#### Domestic violence

Experiences of abuse including physical, sexual, and psychological and controlling behaviors perpetrated by women’s partners and/or his family members were measured using the 63-item Indian Family Violence and Control Scale [IFVCS; (National Family Health Survey, 2017)]. The IFVCS was designed for use in the Indian population, with items being derived from informant and expert interviews with an Indian sample to ensure it captured culturally-specific forms of DV (Kalokhe et al., 2015, 2016). Preliminary validation of the IFVCS suggested that the scale has strong internal consistency, and good concurrent and construct validity (Kalokhe et al., 2016). Cronbach’s alphas were calculated for the current sample, indicating that both the control and abuse subscales had very good internal reliability (0.94 and 0.97 respectively).

The control subscale consisted of 14 items which asked women to rate their access to various freedoms during their entire relationship (e.g., “freedom to spend my own money on personal things”) on a 4-point scale, ranging from 0 (*never*), to 3 (*often*). Total scores for this subscale ranged from 0 to 42, with lower scores indicating lower access to freedom, or more frequent controlling behavior. The 49-item abuse subscale comprised of statements relevant to psychological (22 items), physical (16 items), and sexual violence (11 items) domains and asked women about the frequency of abusive behaviors (e.g., “burnt me or threatened to burn me with a cigarette”) on a 4-point scale, from 0 (*never*) *to* 3 (*about once a month*). Higher scores indicated greater frequency of abuse, with the total possible abuse score ranging from 0 to147.

#### Partner’s patriarchal beliefs

Women’s partner’s patriarchal beliefs were measured using 10 items derived from the 5-item Husband’s Patriarchal Beliefs Scale, which was originally developed by Smith (1990) and later adapted by Ahmed-Ghosh (2004), with the addition of 5 items from the 37-item Patriarchal Beliefs Scale (Yoon et al., 2015). The 10 resultant items captured each of the core dimensions of patriarchal ideology identified by Yoon (Yoon et al., 2015); these include beliefs about the institutional power of men, the inherent inferiority of women, and gendered domestic roles. The scale asked women to rate *their perception* of their partner’s level of agreement to various patriarchal beliefs (e.g., “men are inherently smarter than women”) on a 7-point Likert scale ranging from 1 *(strongly disagree)* to 7 (*strongly agree*). Scores ranged from 10 to 70, with higher scores indicating greater endorsement of patriarchal ideology. Cronbach’s alpha was calculated as.96, indicating this new scale had very high internal consistency.

### Procedure

This study was guided by the WHO’s ethical and safety recommendations for DV research (Ellsberg and Heise, 2002) and received approval from the institutional ethics committee in compliance with American Psychological Association (2017) ethical standards (American Psychological Association, 2017). All persons who saw an advertisement or accessed the online link received a plain language statement, as well as information about DV support services in their country, regardless of whether or not they completed the survey. To protect the safety of participants, a Quick Escape button was programmed into the survey. The survey (in English) was anonymous and took approximately 20 min to complete.

## Results

### Data screening and cleaning

Data cleaning was conducted prior to analysis. Cases missing more than 50% of their data were removed from the sample. For the remaining cases, random missing values were replaced with the series mean. All items across the three abuse subscales, and the control subscale of the IFVCS were summed to obtain a total abuse, and total control score, respectively. For the purposes of regression analyses, employment was dichotomised as employed versus not employed, and education as tertiary education versus non-tertiary education. Each nominal independent variable was treated as a set of dummy variables, with one variable serving as the reference group. For the regression analyses, only women who had reported some form of abuse were included in the analysis; thus, the 15.9% of the sample that reported no abuse were excluded from the analyses.

### Analytical strategy

First, descriptive analyses were undertaken to determine the extent of DV and partners’ patriarchal beliefs in the sample and these are presented in Table 2. As control and abuse were measured on different scales, two hierarchical multiple regression analyses (as seen in Table 3) were conducted to test the central hypothesis. For each regression analysis, a three-stage hierarchical regression, and bottom-up model building strategy was used. In model 1, a univariate model including patriarchal beliefs, and either abuse or control as the outcome measure was tested. This provided a baseline estimation of the variance in abuse or control predicted by patriarchal beliefs, enabling estimation of the contribution of the variables added hierarchically in subsequent models. In Model 2, demographic variables (age, marital status, educational attainment, employment status, migration status, and continent of residence) identified in the literature review as potential confounders were entered into the model; all demographic variables were entered into the model together. Two-way interaction effects between patriarchal beliefs and each of the demographic characteristics were examined to exclude potential moderation effects.

**Table 2:** Descriptive statistics for abuse (*N* = 729), control (*N* = 825) and partners’ patriarchal beliefs (*N* = 729).

Type of DV	*n* (%)	*M*	*SD*
Physical abuse		3.12	5.90
Never	356 (43.2%)		
Ever	374 (45.2%)		
Emotional abuse		9.72	11.40
Never	153 (18.5%)		
Ever	576 (69.9%)		
Sexual abuse		1.17	3.57
Never	550 (66.7%)		
Ever	179 (21.7%)		
Any abuse		13.86	18.14
Never	131 (15.9%)		
Ever	598 (72.5%)		
Any control^a^		32.56	9.12
Ever	290 (35.1%)		
Patriarchal beliefs		26.27	16.28

^a^Due to the scale used for the control subscale, the number of women who were never controlled could not be calculated. High scores on this subscale indicate greater access to freedom.

**Table 3:** Summary of hierarchical regression analysis for variables predicting control (*N* = 579).

	Model 1			Model 2		
Variable	*B*	95% CI	*ß*	*B*	95% CI	*ß*
Patriarchal beliefs	−0.22	[−0.26, 0.17]	−0.38***	−0.20	[−0.24, −0.16]	−0.35***
Age				0.09	[0.01, 0.18]	0.09*
Education						
Non-tertiary				Referent		
Tertiary				1.93	[−1.50, 4.34]	0.05
Employment status						
Unemployed				Referent		
Employed				1.36	[−0.11, 2.82]	0.07
Marital status						
Married				Referent		
Separated				−7.07	[−8.99, −5.18]	−0.28***
Widowed				−3.57	[−9.62, 2.91]	−0.04
*De facto*				1.39	[−1.97, 4.75]	0.03
Continent						
Asia				Referent		
Oceania				−1.81	[−4.10, 0.48]	−0.08
Europe				−1.34	[−3.57, 0.89]	−0.06
Americas				−0.21	[−2.58, 2.15]	−0.01
Africa				2.61	[−1.42, 6.63]	0.05
Migration status						
Never migrated				Referent		
Migrated				−0.69	[−2.61, 1.23]	−0.04
R^2^		0.146		0.249		
Adjusted R^2^		0.144		0.233		
*△*R^2^		0.146		0.103		
*△*F		98.49***		7.04***		
*df △*F		1, 577		11, 566		

***Denotes significance at the 0.01 level; *denotes significance at the 0.05 level.

### Descriptive analyses

#### Abuse and control

Results (seen in Table 2) demonstrated that 72.5% of women reported having experienced at least one instance of abuse in their lifetime, while 15.9% reported no abuse. Across the different subscales, 69.9% had experienced some form of psychological abuse, 45.2% had experienced physical abuse and 21.7% had experienced sexual abuse. Over a third of participants (35.1%) had on at least one occasion had an aspect of their freedom denied by their partner.

#### Patriarchal beliefs

The descriptive statistics for patriarchal beliefs are also presented in Table 2. The Mean scores (*M* = 26.27, *SD* = 16.28) indicated an overall tendency for partners to disagree with patriarchal beliefs.

### Multiple regression analyses

A detailed summary of the hierarchical regression is presented in Table 3.

In Model 1, the univariate model, patriarchal beliefs was associated with a statistically significant 14.4% of the variance in controlling behavior. Women whose partners endorsed stronger patriarchal beliefs had less access to freedom in their relationship (*ß* = −0.38, *p* < 0.001). Introducing demographic variables in Model 2 using the Stepwise method was associated with a statistically significant additional 10.3% of variance in control. Specifically, women experienced significantly more control (<0.05) with increasing age and significantly less control (<0.01) when they were separated compared to women who were married. The beta value for patriarchal beliefs remained statistically significant and largely unchanged with the addition of the demographic variables (*ß = −0.35, p* < 0.001). Patriarchal beliefs alone accounted for 11.49% (*sr^2^* = 0.12) of the total variance in controlling behavior. In addition to patriarchal beliefs, two of the 11 demographic variables were significant predictors of control. Inspection of two-way interaction effects between PBS and each of the demographic characteristics indicated no evidence of moderation occurring. The final model accounted for 23.3% of the variance in control *F* (12, 566) = 15.61, *p* < 0.001, which is considered a large effect (Cohen, 1988).

A detailed summary of the hierarchical regression is presented in Table 4.

**Table 4:** Summary of hierarchical regression analysis for variables predicting abuse (*N* = 577).

	Model 1			Model 2		
Variable	*B*	95% CI	*ß*	*B*	95%CI	*ß*
Patriarchal beliefs	0.39	[0.30, 0.47]	0.34***	0.35	[0.26, 0.40]	0.31***
Age			0.02	−0.03	[−0.21, 0.15]	−0.01
Education						
Non-tertiary				Referent		
Tertiary			−0.04	−1.95	[−8.01, 4.10]	−0.03
Employment status						
Unemployed				Referent		
Employed			−0.04	−0.40	[−3.50, 2.70]	−0.01
Marital status						
Married				Referent		
Separated			0.25***	10.94	[6.91, 14.96]	0.21***
Widowed			−0.03	−5.11	[−18.38, 8.15]	−0.03
*De facto*			−0.06	−4.63	[−11.86, 2.60]	−0.05
Continent						
Asia				Referent		
Oceania			−0.04	−2.23	[−7.10, 2.63]	−0.05
Europe			−0.07	−2.23	[−6.96, 2.50]	−0.05
Americas			−0.09	−3.90	[−8.90, 1.12]	−0.08
Africa			−0.05	−5.64	[−14.18, 2.89]	−0.05
Migration status						
Migrated				Referent		
Never migrated			−0.10	−2.90	[−6.97, 1.18]	−0.08
Adjusted R^2^		0.11		0.155		
F for change in R^2^		75.02***		3.52***		

***Denotes significance at the 0.01 level.

For Model 1, the univariate model, partners’ patriarchal beliefs was associated with a statistically significant 11.4% of the variance in experience of abuse. Women who perceived their partners held stronger patriarchal beliefs were more likely to have been abused (*ß* = 0.34, *p* < 0.001). The addition of demographic variables was associated with a statistically significant additional 5.7% of the variability in abuse (Model 2). This final model explained 15.5% of the variance in abuse, adjusted *R^2^* = 0.155, *F* (12, 564) = 9.79, *p* < 0.001, which is considered a medium effect (Cohen, 1988). The beta value for patriarchal beliefs remained a significant independent predictor of abuse (*ß* = 0.31*, p* < 0.001). Patriarchal beliefs contributed the highest amount of variance in abuse, independently contributing 9% (sr^2^ = 0.09). Inspection of two-way interaction effects between PBS and each of the demographic characteristics indicated no evidence of moderation.

## Discussion

This study is the first to examine the relationship between domestic violence and a partner’s adherence to patriarchal ideology in the global Indian context. The findings support the hypothesis that women who perceived their partners to endorse greater patriarchal beliefs were more likely to have been abused and subjected to controlling behavior.

The finding that partners’ patriarchal beliefs predicted DV victimization lends support to the longstanding feminist propositions that DV occurs mainly in contexts where patriarchal ideologies are dominant (Jejeebhoy and Cook, 1997; Haj-Yahia, 2005; Satyen, 2021). In this study, women who believed that their partners viewed women in general as inherently inferior to men, legitimized male authority in public and private arenas, endorsed prescriptive gender roles, and condoned the use of violence for gender-role violation were more likely to be abused or controlled by their male partners. This finding is consistent with the limited existing studies that have demonstrated the relationship between male patriarchal ideologies and DV perpetration across three countries including the United States (Sugarman and Frankel, 1996; Stith et al., 2004; Haj-Yahia, 2005; Adam and Schewe, 2007; Watto, 2009). By contributing to the understanding of the experiences of Indian women globally, this study highlights the pervasive and enduring negative influence of the patriarchal ideology on women.

The relationship between patriarchal beliefs and DV persisted after controlling for a range of factors such age, educational attainment, marital status, migration status, employment, and geographical location that have been previously used to explain DV victimization in Indian populations [e.g., (Sabri et al., 2014; Gender, 2015; Kalokhe et al., 2018)]. It further emerged as the strongest independent predictor of women’s experiences of both abuse and control. Such a finding cautions against any theory of DV in Indian communities that overlooks or minimizes gender as an explanatory factor. It also suggests that merely focusing on the individual characteristics of DV victims is problematic in that it conceals the ways in which DV is embedded in broader sociocultural structures including the violence committed in childhood by a father [e.g., (Tsirigotis and Łuczak, 2018)]. This finding removes some of responsibility and shame from both victims of DV and from individual cultural groups, by firmly situating their experiences within a patriarchal framework. This finding also has fundamental practical implications for understanding and preventing DV in Indian communities, by identifying patriarchal beliefs and practices as targets for intervention that are amenable to effecting social change in the continuance of DV.

An unexpected finding was that age, educational attainment, marital status, geographical location, migration status, and employment status did not moderate the relationship between patriarchal beliefs and DV experiences. These findings could be considered in light of the universal phenomenon of gendered violence in women and the significant role of patriarchal beliefs. This is in contrast to an intersectional framework (Crenshaw, 1991) which suggests that different social factors interact and intersect with gender oppression to place certain groups of women at increased risk of DV. While it is possible that this finding may be an artefact of the specific sample included in this study, we did not measure structural patriarchy, for example, casteism and classism, which may be a better proxy for the macro-level gender oppressions and inequalities referred to in intersectionality theory (Heise, 1998). In support of this explanation, one salient finding from the present study was that *continent of residence* was not an independent predictor of either abuse or controlling behavior and did not moderate the relationship between patriarchal beliefs and DV. This suggests that patriarchal beliefs can prevail despite structural gains in women’s empowerment or through migrating to more egalitarian locations (Hunnicutt, 2009). However, the findings also demonstrated that women experienced greater controlling behavior as they became older and, in contrast to women who were married, those who were separated experienced less control. The latter findings could relate to lower levels of control because the women had separated from their partner. It is also possible that as women are older, they are more invested in their relationships and less likely to challenge greater levels of control by their partners. In sum, women’s specific social context does not appear to specify the appropriate conditions for the translation of patriarchal ideas about gender relations and, in particular, DV (Yoon et al., 2015). The findings of this study highlight the need to engage with men at the individual level to challenge the patriarchal beliefs and norms that propagate, justify, and excuse DV.

Based on the findings of this study, it is clear that interventions should use a ‘gender transformative’ approach (Gupta and Sharma, 2003) which acknowledges that DV is inherently gendered and a product of patriarchal ideologies. These interventions could be provided in group or individual formats, should be culturally-tailored, and work with men to promote women’s access to authority and decision-making, as well as challenge traditional gender roles and acceptance of DV (Violence against women in Australia An overview of research and approaches to primary prevention, 2017). Encouraging evidence from the international literature suggest that such programs can lead to short-term changes in both attitudes and behavior, including decreased self-reported use of physical, sexual, and psychological DV (Whitaker et al., 2006; Barker et al., 2010). However, the literature does not reveal if such programs have been piloted in Indian communities.

### Limitations

The primary limitations of the current study relate to the sample characteristics and subsequent generalizability of findings. This study used a convenience sample and as such may not adequately represent Indian women across a range of societies. However, the strength is that women from 31 countries took part in the study. Second, the Partner’s Patriarchal Beliefs scale asked women to rate *their* perception of their partner’s beliefs, and therefore may not have accurately reflected men’s ideologies. However, attempting to understand and validate women’s lived experiences and perceptions is important in any feminist enquiry (Yllö and Bograd, 1984) and wives’ accounts of their husband’s behavior have been found to be more accurate than husband’s account of his own behavior (Arias and Beach, 1987). Nevertheless, future research may wish to further establish the validity and psychometric properties of the scale used. Finally, the cross-sectional nature of this study limits the extent to which we can draw conclusions regarding the temporality or causal nature of the observed associations. While theories of patriarchy suggest it fuels DV, it is also plausible that use of DV also strengthens patriarchal beliefs, by further reinforcing a system of male domination and female subordination in the family. Future studies employing a prospective or longitudinal design and representative sample will strengthen the practical significance of the findings described in this study.

### Conclusions and implications for future research

Notwithstanding the aforementioned limitations, this study is novel in showing the effects of individual-level patriarchal beliefs on women’s experiences of both abuse and control using a large, cross-national sample that adjusted for a range of established risk factors and employed a validated, culturally-sensitive measure of DV. The findings raise awareness of the extent of DV in Indian communities and emphasize the need to collectively acknowledge how gender and culture interact to shape women’s experiences of DV. Such an understanding can have far-reaching implications for the reduction and prevention of DV in Indian communities, by providing mental health practitioners, community leaders, policy makers, women’s activists, and the wider community more broadly, a principal target for intervention. Given the observed associations between partners’ patriarchal beliefs and both abuse and controlling behavior, efforts should be targeted at developing culturally-tailored education strategies aimed at challenging men’s enactment of their investment in patriarchy regardless of their social situation, includingtheir education level, religion, and caste.

While this study focused on patriarchal beliefs as an explanatory model for DV, future research may wish to incorporate other theoretical frameworks in order to develop a comprehensive, integrated, ecological theory of DV that considers other individual, interpersonal, and sociocultural factors alongside patriarchal ideology. Furthermore, whilst this study focused on men’s beliefs, women’s perceptual, cognitive, and behavioral responses to DV are also shaped by patriarchal beliefs (Ahmed-Ghosh, 2004). Therefore, future research should examine how patriarchal beliefs influence other DV processes such as reduced help-seeking behavior that place women at further risk of DV; the intersections between the prevalent Indian social contexts of gender, caste, and violence should also be examined – this will enable the more nuanced understanding of whether women from some castes, especially the lower castes are more prone to controlling and abusive behavior than women in the upper castes [see Deshpande (2003) and Khubchandani et al. (2018) for a broad review of the discrimination between people of different castes and the intersections of this with gender in the Indian society]. Finally, given that culturally-diverse groups of women remain underrepresented in the DV literature, future researchers should consider how patriarchal beliefs manifest in other communities to further enhance our understanding of DV and pave the way for the prevention of violence against all women.

## Data availability statement

The datasets presented in this article are not readily available because the data is sensitive by nature and according to the Deakin University Human Research Ethics Committee protocol, we are not allowed to share this data, even in anonymized form. Requests to access the datasets should be directed to lata@deakin.edu.au.

## Ethics statement

The studies involving humans were approved by the Deakin University Human Research Ethics Committee. The studies were conducted in accordance with the local legislation and institutional requirements. The participants provided their written informed consent to participate in this study.

## Author contributions

LS: Conceptualization, Data curation, Formal analysis, Investigation, Methodology, Project administration, Resources, Supervision, Validation, Writing – original draft, Writing – review & editing. MB-I: Data curation, Formal analysis, Investigation, Methodology, Writing – original draft. BR: Data curation, Formal analysis, Validation, Writing – review & editing.
